# Machine learning as an adjunct to expert observation in classification of radiographic knee osteoarthritis: findings from the Hertfordshire Cohort Study

**DOI:** 10.1007/s40520-023-02428-5

**Published:** 2023-05-19

**Authors:** Leo D. Westbury, Nicholas R. Fuggle, Diogo Pereira, Hiroyuki Oka, Noriko Yoshimura, Noriyuki Oe, Sasan Mahmoodi, Mahesan Niranjan, Elaine M. Dennison, Cyrus Cooper

**Affiliations:** 1 MRC Lifecourse Epidemiology Centre, University of Southampton, Southampton, UK; 2 The Alan Turing Institute, London, UK; 3 Departamento de Engenharia Electrotécnica e de Computadores, Faculdade de Ciências e Tecnologia, FCT/UNL, Universidade Nova de Lisboa, 2829-516 Caparica, Portugal; 4 Instituto de Telecomunicacoes, 1049-001 Lisbon, Portugal; 5 Department of Medical Research and Management for Musculoskeletal Pain, 22nd Century Medical and Research Center, The University of Tokyo, Tokyo, 113-8655 Japan; 6 Department of Preventive Medicine for Locomotive Organ Disorders, 22nd Century Medical and Research Center, The University of Tokyo, Tokyo, Japan; 7 Faculty of Engineering and Physical Sciences, Electronics and Computer Science, University of Southampton, Southampton, UK; 8 Victoria University of Wellington, Wellington, New Zealand; 9 NIHR Southampton Biomedical Research Centre, University of Southampton and University Hospital Southampton NHS Foundation Trust, Southampton, UK; 10 NIHR Oxford Biomedical Research Centre, University of Oxford, Oxford, UK

**Keywords:** Epidemiology, Musculoskeletal, Artificial intelligence, Kellgren and Lawrence

## Abstract

**Background:**

Osteoarthritis is the most prevalent type of arthritis. Many approaches exist for characterising radiographic knee OA, including machine learning (ML).

**Aims:**

To examine Kellgren and Lawrence (K&L) scores from ML and expert observation, minimum joint space and osteophyte in relation to pain and function.

**Methods:**

Participants from the Hertfordshire Cohort Study, comprising individuals born in Hertfordshire from 1931 to 1939, were analysed. Radiographs were assessed by clinicians and ML (convolutional neural networks) for K&L scoring. Medial minimum joint space and osteophyte area were ascertained using the knee OA computer-aided diagnosis (KOACAD) program. The Western Ontario and McMaster Universities Osteoarthritis Index (WOMAC) was administered. Receiver operating characteristic analysis was implemented for minimum joint space, osteophyte, and observer- and ML-derived K&L scores in relation to pain (WOMAC pain score > 0) and impaired function (WOMAC function score > 0).

**Results:**

359 participants (aged 71–80) were analysed. Among both sexes, discriminative capacity regarding pain and function was fairly high for observer-derived K&L scores [area under curve (AUC): 0.65 (95% CI 0.57, 0.72) to 0.70 (0.63, 0.77)]; results were similar among women for ML-derived K&L scores. Discriminative capacity was moderate among men for minimum joint space in relation to pain [0.60 (0.51, 0.67)] and function [0.62 (0.54, 0.69)]. AUC < 0.60 for other sex-specific associations.

**Discussion:**

Observer-derived K&L scores had higher discriminative capacity regarding pain and function compared to minimum joint space and osteophyte. Among women, discriminative capacity was similar for observer- and ML-derived K&L scores.

**Conclusion:**

ML as an adjunct to expert observation for K&L scoring may be beneficial due to the efficiency and objectivity of ML.

## Introduction

Osteoarthritis (OA) is the most prevalent type of arthritis and is characterised by joint stiffness and pain, leading to functional decline [1]. The Global Burden of Disease 2017 Study found that OA accounted for 14.9 million incident cases, 303.1 million prevalent cases, and 9.6 million years lived with disability in 2017 [2]. The knee is the most common site of OA, with the prevalence of knee OA estimated at around 50% among those aged 75 years and older [3].

Knee OA can be characterised through use of radiography and clinical information relating to patient-reported symptoms and function [4]. However, previous studies have established discordance between the presence of radiographic and clinical knee OA [5–7] and much interest has focussed on how to characterise radiographic OA. One approach for deciding between different methods is to examine their predictive capacity regarding pain and degree of impaired function, two of the key clinical symptoms of OA [4]. It has been suggested that Kellgren and Lawrence (K&L), with its composite joint space, osteophytes, sclerosis and altered joint congruity, provides a better index than individual radiographic features alone for the prediction of knee pain [8, 9].

Supervised machine learning (ML), the process by which algorithms 'are taught' to recognise labelled data such that they can accurately predict future outcomes from new, unlabelled data, has been widely applied in medical research [10] and in the field of osteoarthritis [11]. There can be wide variation in the subjective assessment of knee radiographs with regard to the K&L grading of osteoarthritis severity [12] which could be avoided by applying ML techniques which also have the potential to improve efficiency by assisting radiologists and radiographers in their assessment of knee radiographs. This is important in the context of a widespread shortage of radiologists; in 2021, the consultant radiologist workforce shortfall stood at 29% (1669 whole-time equivalents) in the UK alone [13].

To our knowledge, no studies have compared how strongly individual radiographic features (minimum joint space and osteophyte), observer-derived K&L scores and ML-derived K&L scores are related to pain and function. Therefore, we explored this in a population-based cohort of community-dwelling older men and women from the United Kingdom.

## Methods

### The Hertfordshire Cohort Study

The Hertfordshire Cohort Study (HCS) comprises men and women born in Hertfordshire from 1931–1939 and who still lived there in 1998–2004 when they completed a clinic visit and home interview for a detailed characterisation of their health. The HCS and further details of the associated follow-up studies have been described in detail previously [14, 15].

### Ascertainment of participant characteristics in 2011

Smoking status, alcohol consumption and average daily outdoor physical activity in minutes (Longitudinal Aging Study Amsterdam Physical Activity Questionnaire (LAPAQ) [16]) were ascertained at the home interview through nurse-administered questionnaires. The Western Ontario and McMaster Universities Osteoarthritis Index (WOMAC), a 24-item knee questionnaire with subscales measuring pain, stiffness and physical function [17], was also administered. Height was measured (wall-mounted SECA stadiometer) along with weight (calibrated SECA 770 digital floor scales, SECA Ltd, Hamburg) and used to derive BMI (kg/m^2^).

Anterior–posterior and lateral patellofemoral knee X-rays were taken of both knees at a local hospital after the 2011 home visit and joints were graded based on the (K&L) criteria [18]. This criteria is described as follows: Grade 1—possible osteophytes on the radiograph and unlikely narrowing of the joint space; Grade 2—small osteophytes and possible narrowing of the joint space; Grade 3—multiple, moderately sized osteophytes, definite joint space narrowing, some sclerotic areas and possible deformation of bone ends; Grade 4—multiple large osteophytes, severe joint space narrowing, marked sclerosis and definite bony end deformity [18].

### Derivation of minimum joint space and osteophyte from radiographs

The automatic knee OA computer-aided diagnosis (KOACAD) program to quantify key OA parameters from digital knee radiographs has been described in detail previously [19]. In brief, filtering of the radiograph was performed to reduce image noise and to extract outlines of the tibia and femur for estimation of medial and lateral sides. Measurements of joint space area and minimum joint space were ascertained after determination of the region of interest. The medial and lateral tibial and femoral margins were then constructed using a horizontal neighbourhood difference filter and Canny’s filter in order to calculate inflection points for these margins. The medial tibial outline from the joint level to the inflection point was then drawn; osteophyte area was regarded as the area that was medially prominent over the extended outline.

### Derivation of K&L grades from machine learning

To develop the machine learning algorithm, data from the Osteoarthritis Initiative (OAI), a prospective observational study of 4796 individuals with, or at risk of, developing knee OA [20], were separated into a training dataset and a validation dataset. The final algorithm was then applied in the Hertfordshire Cohort Study.

To perform the ML, data from HCS participants, comprising knee radiographs and the corresponding K&L grade, were combined with similar data obtained from Mendeley Data [21]. The latter contained 2889 training and 828 testing radiographs from OAI participants.

To detect joints in radiographs, Faster R-CNN was used [22]. This consists of the following process: network filters are convolved with the radiograph to yield two-dimensional feature maps; regions of feature maps are then generated by sliding a window through them; finally, feature vectors corresponding to each region are extracted, from which the probability that the region contains the joint, along with the coordinates of the boundary of the region, are estimated. For the training process, the Mendeley training dataset was used. Radiographs were resized to 320 × 256 and those containing joint replacements were removed. The backbone network used was a ResNet-50 [23]. All network parameters were randomly initialised. The model was then fine-tuned for 10 epochs, using the Adam optimiser [24] with a starting learning rate of 5 × 10^–5^, which was decreased by a factor of 0.1 every 3 epochs. At the end of each epoch, the test data were assessed. Batch sizes of 5 and 1 were used for training and testing, respectively.

For predicting the K&L grade corresponding to each radiograph, ResNet-152, a type of Convolutional Neural Network [25], was used. All detected joints were cropped. The Mendeley dataset was already split into training (5778 knee joints) and testing (1656 knee joints) arms. Each model was run three times for three different seeds (0, 1, 2). Data augmentation was used to enlarge the training data where every image was horizontally flipped and rotated 30°. All models were trained for ten epochs and at the end of each epoch, the test data were assessed. The epoch with the highest level of accuracy was used. A stochastic gradient descent (SGD) optimiser [26] with momentum of 0.9 and with weight decay of 5 × 10^3^ was implemented. Two different learning rate values were used, 1 × 10^–3^ for all networks except the classifier, which used a learning rate of 5 × 10^–2^. The learning rate was decreased by a factor of 0.1 every 2 epochs. The same batch sizes were used as in the detection process.

### Ethical approval and informed consent

The baseline Hertfordshire Cohort Study had ethical approval from the Hertfordshire and Bedfordshire Local Research Ethics Committee and the follow-up had ethical approval from the East and North Hertfordshire Ethical Committees. Investigations were conducted in accordance with the principles expressed in the Declaration of Helsinki.

### Statistical analysis

Analyses were performed at the person-level as WOMAC scores were only available for individual participants and not for each knee. As a result, the worse value from both knees (highest K&L score and osteophyte, and lowest minimum joint space) was used in analyses. Predictors in analyses were: low minimum joint space, defined as having values in the sex-specific lower third of the distribution (< 3.2 mm for men, < 2.8 mm for women); observer-derived and ML-derived K&L scores, categorised as 0/1, 2 and 3/4; and osteophyte, dichotomised as 0 mm^2^ and > 0 mm^2^. Outcomes in analyses were pain (WOMAC pain score > 0) and impaired function (WOMAC function score > 0).

Participant characteristics were described using summary statistics. Predictors in relation to outcomes were examined using chi-squared and Fisher’s exact tests. Logistic regression was used to perform receiver operating characteristic (ROC) analyses to calculate the area under curve (AUC) for each uncategorised predictor in relation to each outcome.

Men and women were analysed separately and P < 0.05 was regarded as statistically significant. Analyses were conducted using Stata, release 17.1.

## Results

### Determination of analysis sample from the Hertfordshire Cohort Study

The Hertfordshire Cohort Study (HCS) comprised 2997 participants at baseline (1998–2004). In 2004, of the 966 participants from East Hertfordshire who had a dual-energy X-ray absorptiometry (DXA) scan at the start of the study, 642 were recruited for a musculoskeletal follow-up study. In 2011, 591 were invited to participate in a further follow-up study; 443 agreed to participate. The analysis sample comprised 359/433 participants with data on at least one key predictor (minimum joint space, osteophyte and ML-derived K&L scores) and at least one outcome (WOMAC scores for pain and impaired function).

### Participant characteristics of the analysis sample

The characteristics of the study population are presented in Table 1. Mean (SD) age at the 2011 follow-up was 75.5 (2.5) years. Mean (SD) minimum joint space was 3.6 (1.0) mm and 3.2 (1.0) mm among men and women, respectively; values for median (lower quartile, upper quartile) osteophyte were 0.8 (0.0, 6.9) mm^2^ among men and 2.1 (0.0, 8.3) mm^2^ among women. Overall, 53 (30.1%) men and 67 (36.6%) women had pain (WOMAC pain score > 0); 57 (34.1%) men and 67 (39.6%) women had impaired function (WOMAC function score > 0).

**Table 1:** Participant characteristics in 2011

Characteristic	Mean (SD); median (lower quartile, upper quartile); or N (%)		Obs
	Men (n = 176)	Women (n = 183)	
Age (years)	75.4 (2.5)	75.7 (2.6)	0
Height (cm)	172.9 (6.4)	158.8 (5.9)	3
Weight (kg)	82.9 (11.8)	72.1 (13.5)	0
BMI (kg/m^2^)	27.8 (3.8)	28.5 (4.9)	3
Current smoking	8 (4.5%)	4 (2.2%)	0
Alcohol consumption (units/week)	6.3 (1.6, 13.0)	0.3 (0.0, 3.1)	0
Physical activity in last 2 weeks (min/day)^a^	190.4 (111.4, 284.3)	205.7 (137.1, 284.3)	24
Minimum joint space (mm)^c^	3.6 (1.0)	3.2 (1.0)	19
Low MJS (M < 3.2 mm, W < 2.8 mm)^b,c^	62 (37.6%)	61 (34.9%)	19
Osteophyte (mm^2^)^d^	0.8 (0.0, 6.9)	2.1 (0.0, 8.3)	19
Osteophyte (> 0 mm^2^)^d^	109 (66.1%)	129 (73.7%)	19
Observer-derived K&L grade^d^			
0/1	107 (60.8%)	104 (57.1%)	1
2	56 (31.8%)	65 (35.7%)	
3/4	13 (7.4%)	13 (7.1%)	
Machine learning K&L grade^d^			
0/1	118 (67.0%)	122 (67.0%)	1
2	46 (26.1%)	38 (20.9%)	
3/4	12 (6.8%)	22 (12.1%)	
Pain (WOMAC pain score > 0)	53 (30.1%)	67 (36.6%)	0
Impaired function (WOMAC function score > 0)	57 (34.1%)	67 (39.6%)	23

*Obs* Number of missing observations, *K&L* Kellgren & Lawrence, *MJS* Minimum joint space, *WOMAC* Western Ontario and McMaster Universities Osteoarthritis Index

^a^Ascertained using the Longitudinal Aging Study Amsterdam Physical Activity Questionnaire

^b^Bottom sex-specific third of the distribution (<3.2 mm (men), <2.8 mm (women))

^c^Lowest value from both knees used

^d^Highest value from both knees used

### Minimum joint space, osteophyte and K&L scores in relation to pain and impaired function

The proportion with pain and impaired function according to each predictor (minimum joint space, osteophyte, observer-derived K&L score and ML-derived K&L score) is presented in Table 2. Among men, the proportion with impaired function was greater among those with low minimum joint space compared to those without (46.6% vs 26.3%, p = 0.009). Among both men and women, observer- and ML-derived K&L scores were associated with both pain and impaired function (p < 0.05 for all associations); higher proportions with pain and impaired function were observed among participants with higher K&L scores. Osteophyte was not related to pain or impaired function among men or women.

**Table 2:** Proportion with pain (WOMAC pain score > 0) and impaired function (WOMAC function score > 0) according to predictor

Predictor	Pain (WOMAC pain score > 0)				Impaired function (WOMAC function score > 0)			
	Men		Women		Men		Women	
	N (%)	P	N (%)	P	N (%)	P	N (%)	P
Low MJS^a,b^								
No	27 (26.2%)	0.141	40 (35.1%)	0.442	26 (26.3%)	0.009	39 (38.2%)	0.760
Yes [< 3.2 mm (M), < 2.8 mm (W)]	23 (37.1%)		25 (41.0%)		27 (46.6%)		24 (40.7%)	
Osteophyte^c^								
0 mm^2^	16 (28.6%)	0.729	18 (39.1%)	0.745	14 (26.4%)	0.165	19 (45.2%)	0.345
> 0 mm^2^	34 (31.2%)		47 (36.4%)		39 (37.5%)		44 (37.0%)	
Observer-derived K&L grade^c^								
0/1	22 (20.6%)	< 0.001	27 (26.0%)	0.001	22 (21.0%)	< 0.001	30 (29.7%)	0.001
2	21 (37.5%)		31 (47.7%)		26 (52.0%)		28 (50.9%)	
3/4	10 (76.9%)		9 (69.2%)		9 (75.0%)		9 (75.0%)	
Machine learning K&L grade^c^								
0/1	30 (25.4%)	0.002	33 (27.0%)	< 0.001	35 (31.0%)	0.046	33 (28.2%)	< 0.001
2	14 (30.4%)		20 (52.6%)		14 (33.3%)		18 (56.3%)	
3/4	9 (75.0%)		14 (63.6%)		8 (66.7%)		16 (84.2%)	

*K&L* Kellgren & Lawrence, *MJS* minimum joint space, *WOMAC* Western Ontario and McMaster Universities Osteoarthritis Index, *P* P values correspond to chi-squared or Fisher's exact tests

^a^Bottom sex-specific third of the distribution (<3.2 mm (men), <2.8 mm (women))

^b^Lowest value from both knees used

^c^ Highest value from both knees used

### Receiver operating characteristic analysis for each predictor with pain and impaired function as outcomes

**Fig. 1:**
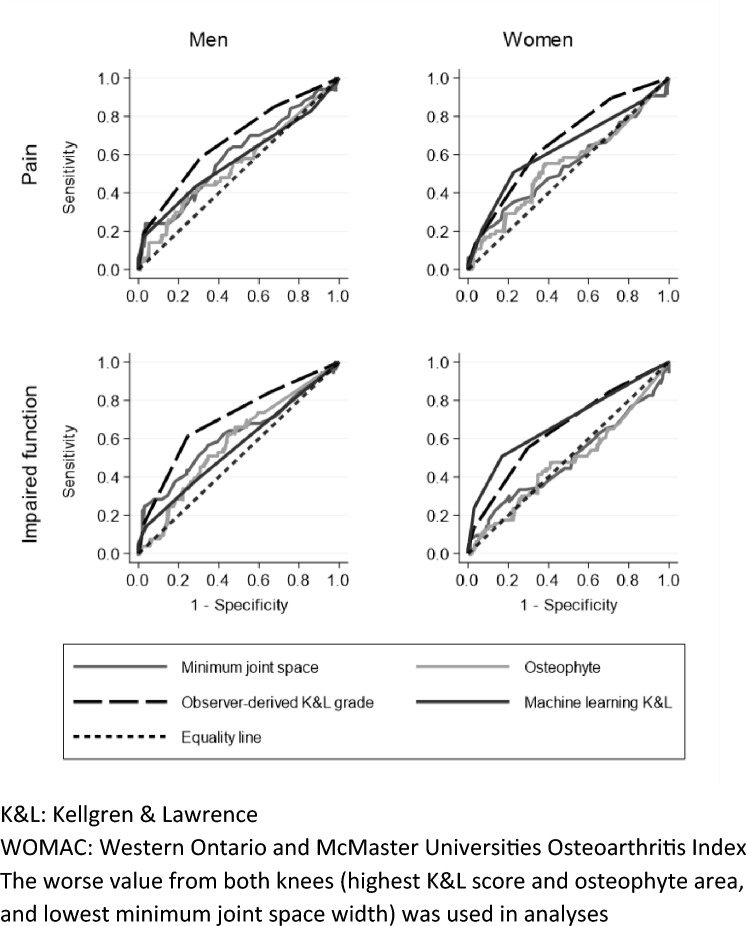
Receiver operating characteristic curves with pain (WOMAC pain score > 0) and impaired function (WOMAC function score > 0) as outcomes. *K&L* Kellgren & Lawrence, *WOMAC* Western Ontario and McMaster Universities Osteoarthritis Index. The worse value from both knees (highest K&L score and osteophyte area, and lowest minimum joint space width) was used in analyses

The AUCs for each predictor (minimum joint space, osteophyte, observer-derived K&L score and ML-derived K&L score) in relation to pain and impaired function as outcomes are presented in Table 3 and Fig. 1. Among men and women, discriminative capacity regarding pain and impaired function was fairly high for observer-derived K&L scores with AUCs ranging from 0.65 (95% CI 0.57, 0.72) to 0.70 (0.63, 0.77), depending on the outcome and whether the subsample comprised men or women; this was only the case among women for ML-derived K&L scores with AUCs of 0.63 (0.56, 0.70) and 0.68 (0.61, 0.75) for pain and impaired function, respectively. Discriminative capacity was moderate among men for minimum joint space in relation to pain [0.60 (0.51, 0.67)] and impaired function [0.62 (0.54, 0.69)]. All other sex-specific associations, including those for osteophyte, had AUCs of less than 0.60.

**Table 3:** Receiver operating characteristic analysis with pain (WOMAC pain score > 0) and impaired function (WOMAC function score > 0) as outcomes

Predictor	Area under curve (AUC)			
	Pain (WOMAC pain score > 0)		Impaired function (WOMAC function score > 0)	
	Men	Women	Men	Women
Minimum joint space^a^	0.60 (0.51, 0.67)	0.54 (0.47, 0.62)	0.62 (0.54, 0.69)	0.49 (0.41, 0.57)
Osteophyte^b^	0.55 (0.47, 0.63)	0.54 (0.47, 0.62)	0.58 (0.50, 0.66)	0.48 (0.41, 0.56)
Observer-derived K&L grade^b^	0.68 (0.60, 0.74)	0.67 (0.60, 0.74)	0.70 (0.63, 0.77)	0.65 (0.57, 0.72)
Machine learning K&L grade^b^	0.57 (0.50, 0.65)	0.63 (0.56, 0.70)	0.56 (0.48, 0.64)	0.68 (0.61, 0.75)

*K&L* Kellgren & Lawrence, *WOMAC* Western Ontario and McMaster Universities Osteoarthritis Index

^a^Lowest value from both knees used

^b^Highest value from both knees used

## Discussion

In this study, K&L assessment by expert observer had higher discriminative capacity regarding WOMAC pain and function compared to minimum joint space and osteophyte, derived from the automatic KOACAD program. For example, AUCs (95% CI) for pain and impaired function ranged from 0.65 (95% CI 0.57, 0.72) to 0.70 (0.63, 0.77) for observer-derived K&L scores among both men and women. In contrast, AUCs for minimum joint space among men were 0.60 (0.51, 0.67) and 0.62 (0.54, 0.69) for pain and impaired function, respectively, with other associations for minimum joint space and osteophyte having AUCs of less than 0.60. To our knowledge, no studies have compared minimum joint space and osteophyte, assessed using the KOACAD system, against observer-defined K&L scores regarding their strength of association with WOMAC pain and function. However, a previous study examined clinical OA (knee pain plus crepitus) in relation to the severity of osteophytes, joint space narrowing and K&L scores (all assessed qualitatively from radiographs) among participants of the Framingham Osteoarthritis Study [9]. Similar to our findings, this study reported that efficiency ([sensitivity + specificity]/2) was highest for K&L scores, suggesting that these should be preferentially deployed in clinical practice.

Our study illustrates that automatic K&L scoring from radiographs can be performed using ML. This may offer advantages such as a reduction in the time required for K&L assessment, reducing the burden on the radiology workforce, and the avoidance of observer-dependent subjectivity. However, K&L assessment by ML did not perform as well as K&L assessment by expert observer in the prediction of the clinical variables of pain and function. Whilst AUCs for ML-derived K&L scores were similar to observer-derived scores among women for pain and impaired function, they were lower among men for pain [0.57 (0.50, 0.65) vs 0.68 (0.60, 0.74)] and impaired function [0.56 (0.48, 0.64) vs 0.70 (0.63, 0.77)]. However, these inconsistencies could be due to the fairly small sample of HCS participants used in the analysis.

Previous studies have used ML to assess knee OA severity by automatically estimating K&L scores from radiographs [27–31]. These studies applied convolutional neural networks to images from the OAI or Multicenter Osteoarthritis Study (MOST) and achieved a classification accuracy of 63% to 78% for uncategorised ML-derived K&L scores in relation to uncategorised observer-derived K&L scores. In our sample, the classification accuracy for this was lower at 50% with a Matthew’s correlation coefficient [32] of 0.27, perhaps due to the fairly small sample size. However, these other studies treated the observer-derived K&L scores as the gold standard even though assigning K&L scores is subjective, reflected in the high level of disagreement between radiographers [33–35]. In light of this, our study compared the ML- and observer-derived K&L scores by examining them each in relation to WOMAC pain and function.

We found that discriminative capacity was moderate among men for minimum joint space in relation to pain and impaired function (AUCs: 0.60–0.62) but weaker for osteophyte. In agreement with these findings, knee pain was more strongly associated with minimum joint space than osteophyte in an analysis of 1001 Japanese participants from the Research on Osteoarthritis Against Disability (ROAD) study which also used the KOACAD system: minimum joint space was associated with knee pain after adjustment for potential confounders but associations regarding osteophyte were not statistically significant [19]. However, an analysis of 2039 participants from the same cohort reported that minimum joint space was significantly associated with WOMAC pain, and osteophyte was significantly associated with impaired WOMAC function after adjustment for age and BMI [36]. Moreover, a longitudinal study comprising 1525 ROAD participants found that among men, osteophyte area was an independent predictor of WOMAC pain and impaired function at the 3-year follow-up but minimum joint space was not; among women, minimum joint space was an independent predictor of these outcomes but osteophyte area was not [37]. These differences in findings could be due to differences in adjustments used, whether studies were longitudinal or cross-sectional, or the fact that some studies analysed knees as individual units whereas others regarded the knee with the lowest minimum joint space as the designated knee for each participant.

Our study has some limitations. Firstly, a healthy participant effect is, unsurprisingly, evident in HCS [14] and sample attrition across the various follow-up waves could have resulted in further selection effects. However, the cohort has been shown to be broadly comparable with participants in the nationally representative Health Survey for England [14]. Furthermore, substantial bias would only have been introduced if associations of interest differed markedly between those who participated in comparison with those who were invited to participate but chose not to; this seems unlikely. Secondly, the sample size for this study was fairly small (n = 359). However, our main findings are biologically plausible and similar to those of previous studies. Thirdly, WOMAC scores were only available for individual participants and not for each knee; the worse value from both knees (highest K&L score and osteophyte, and lowest minimum joint space) was used in analyses. This may have led to an underestimation in the magnitude of the reported associations. Finally, machine learning was only performed with the ResNet family of architectures. However, this is a fairly stable architecture that has the advantage of skip connections between layers, which enables efficient gradient propagation during training. While there is a wide range of architectures in computer vision literature, some of which outperform ResNet in tasks considered in that field, we note that those architectures use millions of images for training and are not appropriate for our study. Furthermore, in a recent study by Matsoukas et al., comparing four different architectures on five different medical inference problems, ResNet achieved competitive performance [38]. Strengths of this study are that the HCS has been phenotyped according to strict protocols by highly-trained fieldworkers and managed by an experienced multi-disciplinary team.

In conclusion, observer-derived K&L scores had higher discriminative capacity for pain and function compared to minimum joint space and osteophyte, derived from the automatic KOACAD program. Among women, discriminative capacity was similar for observer- and ML-derived K&L scores. ML as an adjunct to expert observation in the classification of K&L scores may be beneficial due to the efficiency and objectivity of this method, though further work is required.

## Data Availability

Hertfordshire Cohort Study data are accessible via collaboration. Initial enquires should be made to EMD. Potential collaborators will be sent a collaborators’ pack and asked to submit a detailed study proposal to the HCS Steering Group.
